# Regulation of IgM^+^ B Cell Activities by Rainbow Trout APRIL Reveals Specific Effects of This Cytokine in Lower Vertebrates

**DOI:** 10.3389/fimmu.2018.01880

**Published:** 2018-08-13

**Authors:** Irene Soleto, Esther Morel, Diana Martín, Aitor G. Granja, Carolina Tafalla

**Affiliations:** Fish Immunology and Pathology Laboratory, Center for Animal Health Research (CISA-INIA), Madrid, Spain

**Keywords:** teleost fish, APRIL, B cells, IgM, proliferation, MHC II, antigen presentation, BAFF

## Abstract

Tumor necrosis factor ligand superfamily members such as B cell activating factor (BAFF) and a proliferation-inducing ligand (APRIL) have been identified in mammals as key regulators of B cell homeostasis and activation. However, the immune functions of APRIL are not as well defined as those of BAFF. Furthermore, while BAFF is present in all vertebrates, APRIL is missing in some animal groups, suggesting that BAFF has compensated the functions of APRIL in these species. In this context, we thought of great interest to explore the effects of APRIL on teleost B cells, given that APRIL appears for the first time in evolution in bony fish. Thus, in this study, we have performed an extensive analysis of the effect of APRIL on B cells using rainbow trout (*Oncorhynchus mykiss*) as a model species. Our results demonstrate that APRIL induces a specific proliferation of IgM^+^ B cells by itself and increases IgM secretion without promoting a terminal differentiation to plasma cells. APRIL also increased the levels of surface MHC II and augmented the capacity of these cells to process antigen, effects that were exclusively exerted on IgM^+^ B cells. Although our results point to a highly conserved role of APRIL on B cell homeostasis and activation throughout evolution, some specific differential effects have been observed in fish in comparison to the effects of APRIL previously described in mammals. Finally, the effects that APRIL induces on rainbow trout IgM^+^ B cells described in this paper have been compared with those previously reported in response to BAFF.

## Introduction

B cell activating factor (BAFF) and a proliferation-inducing ligand (APRIL), members of the tumor necrosis factor (TNF) family, are cytokines with a key role in B cell homeostasis, activation, and differentiation processes in mammals (1). Both of them are produced as type II transmembrane proteins which are later proteolytically cleaved at a multibasic motif (2, 3). While BAFF is released from the cell surface by processing of its membrane-bound form, APRIL is processed intracellularly by a furin convertase prior to its secretion, thus acting mainly as a secreted factor (2, 3). In contrast to this general rule, some subsets of mammalian macrophages have been shown to express APRIL on the cell surface (4). Concerning the receptors through which they signal, BAFF binds with strong affinity to transmembrane activator and calcium modulator and cyclophilin ligand interactor (TACI, also known as TNFRSF13B) and BAFF receptor (BAFF-R, also known as BR3 or TNFRSF13C) and with lower affinity to B cell maturation antigen (BCMA, also known as TNFRSF17). APRIL, on the other hand, signals through TACI and BCMA (1). Interestingly, APRIL also interacts with the polysaccharide side chains of heparan sulfate proteoglycans, structurally unrelated to TNF receptors (5). All these receptors are preferentially expressed in B cells and their expression profile significantly varies depending on the B cell subset, the anatomical location or the stage of differentiation (6, 7), thus conditioning the response of these cells to BAFF and/or APRIL.

In mammals, BAFF and APRIL are produced by various cell types, which mainly include macrophages/monocytes, DCs and activated T cells (8, 9). In these species, BAFF has been extensively characterized as a key survival and maturation factor for B cells since BAFF-deficient mice suffer an almost complete loss of mature B cells, and consequently have severely decreased humoral responses (10, 11). Similarly, mice overexpressing BAFF suffer a pronounced hypergammaglobulinemia and splenomegalia due to a prolonged survival and hyperactivity of B cells (12). Much less information is available, however, regarding the role of APRIL. APRIL was originally described as a tumor-stimulating growth factor (13). Concerning its effects on B cells, *in vitro* studies have shown that APRIL synergizes with B cell receptor (BCR) signaling to induce B cell proliferation (14). APRIL has also been shown to upregulate antigen-presenting functions of B cells (15). Surprisingly, in mice, APRIL deficiency has no effect in the number of mature B cells as the B cell development in these animals seems normal (16). These results suggest that, *in vivo*, many effects of BAFF can make up to for the lack of APRIL. However, some specific effects are observed in APRIL-deficient mice. These include an increased production of serum IgA in response to mucosally administered T independent (TI) antigens (16), which seems to indicate that APRIL plays an important role in IgA antibody responses. Furthermore, these APRIL-deficient mice exhibited significantly increased serum IgG antibody responses and had increased numbers of germinal centers (GCs) in their spleens after immunization with T dependent (TD) antigens (16), suggesting that APRIL somehow downregulates TD responses while it favors TI B cell responses.

Although BAFF homologs have been identified in many cartilaginous and bony fish [summarized in Ref. (17)], only a few APRIL homolog sequences have been found in teleost, specifically in zebrafish (*Danio rerio*) (18, 19), channel catfish (*Ictalurus punctatus*) (18), Atlantic salmon (*Salmo salar*) (18), rainbow trout (*Oncorhynchus mykiss*) (18), and grass carp (*Ctenopharyngodon idella*) (20). Interestingly, while BAFF is present in all vertebrates, APRIL is missing in cartilaginous fish, birds and several bony fish, suggesting that in some animal groups BAFF might have functionally compensated the loss of APRIL (21).

Concerning its transcriptional regulation, previous studies performed in grass carp have shown that APRIL transcripts are predominantly expressed in the skin, spleen, and anterior kidney in homeostasis (20). These levels of expression were significantly upregulated in most immune tissues in response to a bacterial or a viral infection (20), pointing to an important role of teleost APRIL in the initial stages of antiviral and antibacterial responses. Similarly, an infection with viral hemorrhagic septicemia virus induced the transcription of APRIL in rainbow trout peritoneal IgM^+^ B cells along with that of TACI (22). In addition, the APRIL mRNA levels were shown to be significantly upregulated upon a natural infection with the myxozoan parasite *Tetracapsuloides bryosalmonae*, the causative agent of proliferative kidney disease (23). Interestingly, in this study, the levels of APRIL mRNA significantly correlated with disease progression. At a functional level, recombinant APRIL has been shown to bind and promote the survival of zebrafish splenocytes (18, 19). In addition, experiments in rainbow trout performed by our group demonstrated an increased survival of peritoneal IgM^+^ B cells in response to APRIL (22). Finally, treatment of rainbow trout kidney leukocytes with recombinant APRIL has been shown to upregulate IgM transcription (23).

Despite these few functional studies, to date, the precise role of APRIL in B cell functionality remains largely unknown in fish, as it is also in mammals, and this is what we have addressed in this study. Our results demonstrate that rainbow trout APRIL is a strong inducer of splenic IgM^+^ B cell proliferation, unlike BAFF which exclusively augments IgM^+^ B cell survival without exerting lymphoproliferative effects (24). In addition, APRIL increased IgM secretion and cell size, but without provoking an apparent terminal differentiation to plasma cells given that the Blimp1 transcription levels were not significantly induced. Finally, APRIL also increased the expression of surface MHC II and the antigen-processing capacities of IgM^+^ B cells, suggesting a specific activation of the antigen-presenting properties of these cells in response to APRIL, not observed for IgM^−^ lymphocyte subsets. These results provide relevant information to understand the role of this cytokine on B cell functionality in fish, helping us to interpret the evolutionary relations between molecules of the APRIL/BAFF family. In this sense, similarities and differences between the effects that BAFF and APRIL exerted in teleost B cells are also discussed in this article.

## Materials and Methods

### Experimental Fish

Healthy specimens of female rainbow trout (*O. mykiss*) of approximately 50–70 g were obtained from Centro de Acuicultura El Molino (Madrid, Spain). Fish were maintained at the Animal Health Research Center (CISA-INIA) laboratory at 16°C with a re-circulating water system and 12:12 h light:dark photoperiod. Fish were fed twice a day with a commercial diet (Skretting, Spain). Prior to any experimental procedure, fish were acclimatized to laboratory conditions for 2 weeks and during this period no clinical signs were ever observed. The experiments described comply with the Guidelines of the European Union Council (2010/63/EU) for the use of laboratory animals and were previously approved by the Ethics committee from the Instituto Nacional de Investigación y Tecnología Agraria y Alimentaria (INIA; Code CEEA PROEX002/17).

### APRIL Transcription in Rainbow Trout Tissues

Rainbow trout were killed by benzocaine (Sigma-Aldrich) overdose and blood was extracted with a heparinized needle from the caudal vein and diluted 10 times with Leibovitz medium (L-15, Invitrogen) supplemented with 100 IU/ml penicillin together with 100 µg/ml streptomycin (P/S, Life Technologies), 10 U/ml heparin (Sigma-Aldrich), and 5% fetal calf serum (FCS, Life Technologies). Peripheral blood leukocytes (PBLs) were obtained from centrifugation (500 × *g* for 30 min at 4°C) of diluted blood on 51% continuous Percoll (GE Healthcare) density gradients. A transcardial perfusion of the rainbow trout was performed using Ringer solution pH 7.4 containing 0.1% procaine to remove blood from fish tissues. Adipose tissue, gonad, brain, foregut, stomach, pyloric caeca, midgut, hindgut, heart, spleen, skin, gills, anterior and posterior kidney, liver, and thymus samples were then collected and placed in Trizol (Thermo Fisher Scientific). DNase I-treated total RNA was prepared from tissue samples or PBLs using a combination of Trizol (Invitrogen) and an RNAeasy Mini kit (Qiagen) as described previously (25). Total RNA was eluted from the columns in RNase-free water, quantified using a Nanodrop 1000 spectrophotometer (Thermo Fisher Scientific) and stored at −80°C until use. For each sample, 2 µg of total RNA was reverse transcribed using Bioscript reverse transcriptase (Bioline Reagents Ltd.) primed with oligo (dT)_12–18_ (0.5 µg/ml), following the manufacturer’s instructions. cDNA was diluted in nuclease-free water and stored at −20°C.

To evaluate the levels of APRIL transcription, real-time PCR was performed in a LightCycler 96 System instrument (Roche) using FastStart Essential DNA Green Master reagents (Roche) and specific primers (Table S1 in Supplementary Material) as previously described (23). Each sample was measured in duplicate under the following conditions: 10 min at 95°C, followed by 40 amplification cycles (30 s at 95°C and 1 min at 60°C). The levels of APRIL expression were normalized to those of trout EF-1α and expression levels calculated using the 2^−ΔCt^ method, where ΔCt is determined by subtracting the EF-1α value from the target Ct as described previously (26, 27). Negative controls with no template and *minus* reverse transcriptase controls [−room temperature (RT)] were included in all experiments.

### Transcriptional Analysis of Isolated Leukocyte Populations

Single cell suspensions from spleen and gills were prepared using 100-µm nylon cell strainers (BD Biosciences) and L-15 medium supplemented with antibiotics (P/S) and 5% FCS. Skin cell suspensions were also prepared. For this, prior to cell extraction, pieces of skin were incubated for 30 min at 4°C in L-15 medium with antibiotics (P/S) and 5% FCS, followed by agitation for 30 min in PBS containing 1 mM EDTA and 1 mM DTT. Tissue digestion was performed using 0.15 mg/ml collagenase type IV from *Clostridium histolyticum* (Sigma) in L-15 for 1.5 h at 20°C. All cell suspensions were placed onto 30/51% Percoll density gradients and centrifuged at 500 × *g* for 30 min at 4°C. Cells at the interface were collected and washed twice in L-15 medium containing 5% FCS.

The constitutive levels of APRIL transcription were studied in IgM^+^ B cells and T cells from spleen as well as from CD8^+^ dendritic cells (CD8^+^ DCs) found in skin and gills after isolating the cells following the methods previously established (23, 28). The expression levels of Blimp-1, CD80/86, CD83, and CD40 were also analyzed on IgM^+^ B cells from spleen using specific primers previously described (Table S1 in Supplementary Material). For this, DNase I-treated total RNA was reverse transcribed directly from FACS sorted populations using the Power Sybr Green Cells-to-Ct Kit (Invitrogen) following the manufacturer’s instructions. For comparative purposes, RNA was also isolated from the RTS11 rainbow trout macrophage–monocyte cell line (29). Real-time PCR was performed using SYBR Green PCR core Reagents (Applied Biosystems) using specific primers and following the manufacturer’s instructions as described previously (30).

### APRIL Transcription at Early Life Stages

To investigate whether APRIL is expressed at early life stages, eyed eggs at different degree days (DD) post-fertilization (~306 DD, ~354 DD, and ~402 DD), immediate post hatch fry (hatch, ~450 DD), pre-first feeding fry (PFF, ~562 DD), fry at the stage of full disappearance of the yolk sac (first feeding, FF, ~674 DD), and fry 3 weeks following first feeding (Fry, 786 DD) were sampled. The fish were maintained at 16°C in recirculated freshwater. Total RNA was extracted and cDNA prepared for real-time PCR analysis from eggs or whole fry using a combination of Trizol (Invitrogen) and an RNAeasy Mini kit (Qiagen) as described above for the analysis of gene expression on tissues.

### Production of Recombinant Rainbow Trout APRIL

The nucleotide sequence corresponding to the extracellular domain of the rainbow trout APRIL sequence (GenBank Accession number NP_001118143.1) together with an N-terminal 6× histidine tag was synthetized and subcloned into the E3 expression vector (Abyntek). The recombinant plasmid was transformed into BL21 cells and a kanamycin-resistant single-positive colony was then incubated at 37°C in Luria–Bertan media. When the OD_600_ reached 0.6, 0.1 mM of isopropyl β-d-thiogalactoside (IPTG, Sigma-Aldrich) was added to induce protein production. After 16 h, cells were harvested, lysed by sonication, and dissolved using urea. Thereafter, APRIL was obtained through the use of Nickel columns (Sigma-Aldrich). The APRIL-containing fractions were pooled and refolded. For the refolding, 0.2% sodium lauroyl sarcosine was added to 4.5 ml of APRIL, and the protein was then dialyzed into 450 ml of 50 mM Tris–HCl, 150 mM NaCl, 10% glycerol, 2 mM DTT, 0.2% sodium lauroyl sarcosine, and pH 8.0. The dialysis was performed for 4 h using a 14-kDa cutoff dialysis membrane. At this point, the buffer was changed and the protein was dialyzed for an additional 16 h. After dialysis, the sample was centrifuged at 13,000 rpm for 30 min and filtered through a 0.22-µm filter. Protein concentration was determined in a BCA protein assay (Thermo Fisher Scientific) and the recombinant rainbow trout APRIL (0.9 mg/ml) was aliquoted and stored at −80°C until use. To verify the correct folding of the recombinant APRIL, 2 µg of the protein was analyzed by polyacrylamide gel electrophoresis (PAGE) under native or reducing conditions, followed by silver staining. Briefly, gels were fixed in 50% methanol plus 10% acetic acid, sensitized with 0.02% sodium thiosulfate, stained with 0.2% silver nitrate, and developed with 2% sodium carbonate plus 0.04% formaldehyde. Gel images were acquired in a ChemiDoc imaging system with Image Lab Touch software (Bio-Rad). An irrelevant protein with a similar molecular weight to that of recombinant APRIL (24.3 kDa), also bearing an N-terminal His tag was produced in the same conditions and was used as a functional control (C-His).

### Flow Cytometry Analysis

Throughout the experiments, splenocytes (2 × 10^6^ cells/ml) in 96-well plates (100 µl/well) were incubated with recombinant rainbow trout APRIL at a final concentration of 1 µg/ml, with the same concentration of C-His or left unstimulated (control). A wide range of APRIL doses had been initially tested to select the optimal dose on the basis of its effect on B cell survival (data not shown).

The anti-trout IgM (1.14 mAb mouse IgG_1_ coupled to FITC) and the anti-trout MHC II β-chain (mAb mouse IgG_1_ coupled to allophycocyanin, 2 µg/ml) used in this study have been previously characterized (28, 31). Spleen leukocytes were incubated with specific antibodies in with L-15 medium containing P/S and 2% FCS for 30 min, washed three times, and analyzed. In all cases, isotype controls for mouse mAbs (BD Biosciences) were tested in parallel to discard unspecific binding of the Abs. All the incubations were performed at 4°C. During the setting up of the experiments, cell viability was checked using DAPI (0.2 µg/ml). Cell viability was always higher than 95% in our experimental conditions.

### APRIL-Binding Assay

Recombinant trout APRIL was biotinylated using the Lightning-Link^®^ Rapid Biotin kit (Expedeon Inc.) following the manufacturer’s protocols. This biotinylated version of APRIL was used to determine its binding to rainbow trout leukocytes. Splenocytes (2 × 10^6^ cells/ml) isolated as described above were dispensed in 96-well plates (100 µl/well) and incubated with 1 µg/ml of biotinylated APRIL or 1 µg/ml of biotynilated C-His protein for 15 min at 4°C. Thereafter, the cells were washed with L-15 medium containing P/S and 2% FCS and labeled with streptavidin coupled to fluorescein (FITC) (Thermo Fischer Scientific) (1 µg/ml) together with an anti-IgM (1.14 mAb mouse IgG1 coupled to allophycocyanin) (1 µg/ml) mAb for 30 min at 4°C. Thereafter, the cells were washed again and analyzed by flow cytometry on a FACS Celesta flow cytometer (BD Biosciences) equipped with FACS DIVA software. Flow cytometry analysis was performed with Flow Jo 10 (Tree Star).

### Confocal Microscopy

To further investigate the capacity of APRIL to bind trout IgM^+^ B cells, splenocytes were incubated with 1 µg/ml of recombinant biotinylated APRIL in L-15 media supplemented with 5% FCS. After 15 min at 20°C, the cells were washed with serum-free L-15 medium, seeded on poly l-lysine coated slides, and incubated at 20°C for 30 min. After gently washing with PBS, the slides were fixed in 4% PFA for 15 min at RT and then incubated for 1 h at RT with blocking solution (PBS, pH = 7.5, containing 0.01% BSA, 0.02% Tween-20, and 0.5% saponin). Fixed cell slides were then incubated with APC-anti-IgM mAb (1 µg/ml) and FITC-streptavidin (0.5 µg/ml) for 1 h. Samples were counterstained with 1 µg/ml DAPI (Sigma). Laser scanning confocal microscopy images (0.3-µm thickness) were acquired with an inverted Zeiss Axiovert LSM 880 microscope. Images were analyzed with Zen 2.0 (Carl Zeiss) and Fiji (NIH) software packages.

### B Cell Proliferation

The Click-IT EdU Alexa Fluor 488 Flow Cytometry Assay Kit (Life Technologies) was used to measure the proliferation of IgM^+^ B cells in response to APRIL. To carry this out, splenocytes at a concentration of 2 × 10^6^ cells/ml were dispensed in 96-well plates (100 µl/well) and incubated for 3 days at 20°C with APRIL as described above, with the same concentration of C-His or left unstimulated (control). EdU (1 µM) was then added to the cultures and the cells were incubated for an additional 24 h. At this point, the cells were collected and stained with allophycocyanin-anti-IgM mAb (1 µg/ml). Briefly, to analyze incorporation of EdU, cells were then fixed and permeabilized with Cytofix/Cytoperm buffer for 15 min at RT. Finally, the incorporation of EdU to the DNA was detected following the manufacturer’s instructions and then analyzed by flow cytometry.

### ELISPOT Analysis

ELISPOT was used to quantify the number of IgM-secreting B cells. Splenocytes (2 × 10^6^ cells/ml) were dispensed in 96-well plates (100 µl/well) and incubated with APRIL (1 µg/ml), with the same concentration of C-His or left unstimulated (control). After 48 h of incubation at 20°C, cells were transferred to pre-coated ELISPOT plates. To this end, ELISPOT plates containing Inmobilon-P membranes (Millipore) were activated with 70% ethanol for 30 s, coated with anti-trout IgM mAb (clone 4C10) at 2 µg/ml in PBS, and incubated overnight at 4°C. To block non-specific binding to the membrane, plates were then incubated with 2% BSA in PBS for 2 h at RT. Thereafter, leukocytes from individual fish were added to the wells in triplicate at a concentration of 5 × 10^4^ cells/well. After 24 h of incubation at 20°C, cells were washed away five times with PBS and plates were blocked again with 2% BSA in PBS for 1 h at RT. After blocking, biotinylated anti-trout IgM mAb (clone 4C10) was added to the plates and incubated at 1 µg/ml for 1 h at RT. Following additional washing steps (five times in PBS) the plates were developed using streptavidin-HRP (Thermo Fisher Scientific) at RT for 1 h, washed again with PBS and incubated with 3-amino 9-ethylcarbazole (Sigma-Aldrich) for 30 min at RT in the dark. The substrate reaction was stopped by washing the plates with tap water. Once the membranes were dried, the number of spots in each well was determined using an AID iSpot Reader System (Autoimmun Diagnostika GMBH).

### Antigen-Processing Assay

The antigen-processing capacity of IgM^+^ B cells was measured using the EnzChek protease Assay kit (Invitrogen). Briefly, splenocytes at a concentration of 2 × 10^6^ cells/ml, seeded in 96-well plates (100 µl/well), were incubated with recombinant APRIL (1 µg/ml), with the same concentration of C-His or left unstimulated (control) during 48 h at 20°C. After this time, the cells were incubated with green fluorescent BODIPY DQ-CASEIN at 5 µg/ml during 1 h. BODIPY DQ-CASEIN is a self-quenched form of fluorescently labeled CASEIN (32), commonly used to study protease-mediated antigen processing due to the fact that it exhibits bright green fluorescence upon proteolytic processing due to the released dye molecules (33). Afterward, the cells were washed with FACS staining buffer three times and labeled with allophycocyanin-anti-IgM mAb (1 µg/ml) for 30 min at 4°C, washed again, and analyzed by flow cytometry as described above.

### Statistical Analysis

Statistical analyses were performed using a two-tailed Student’s *t*-test with Welch’s correction when the *F* test indicated that the variances of both groups differed significantly. The differences between the mean values were considered significant on different degrees, where * means *p* ≤ 0.05, ** means *p* ≤ 0.01, and *** means *p* ≤ 0.005.

## Results

### APRIL Transcription in Physiological Conditions

Before characterizing the biological functions of rainbow trout APRIL, we performed several transcriptional studies to establish which tissues and leukocyte subsets were producing APRIL in physiological conditions, as well as throughout development.

**Figure 1:**
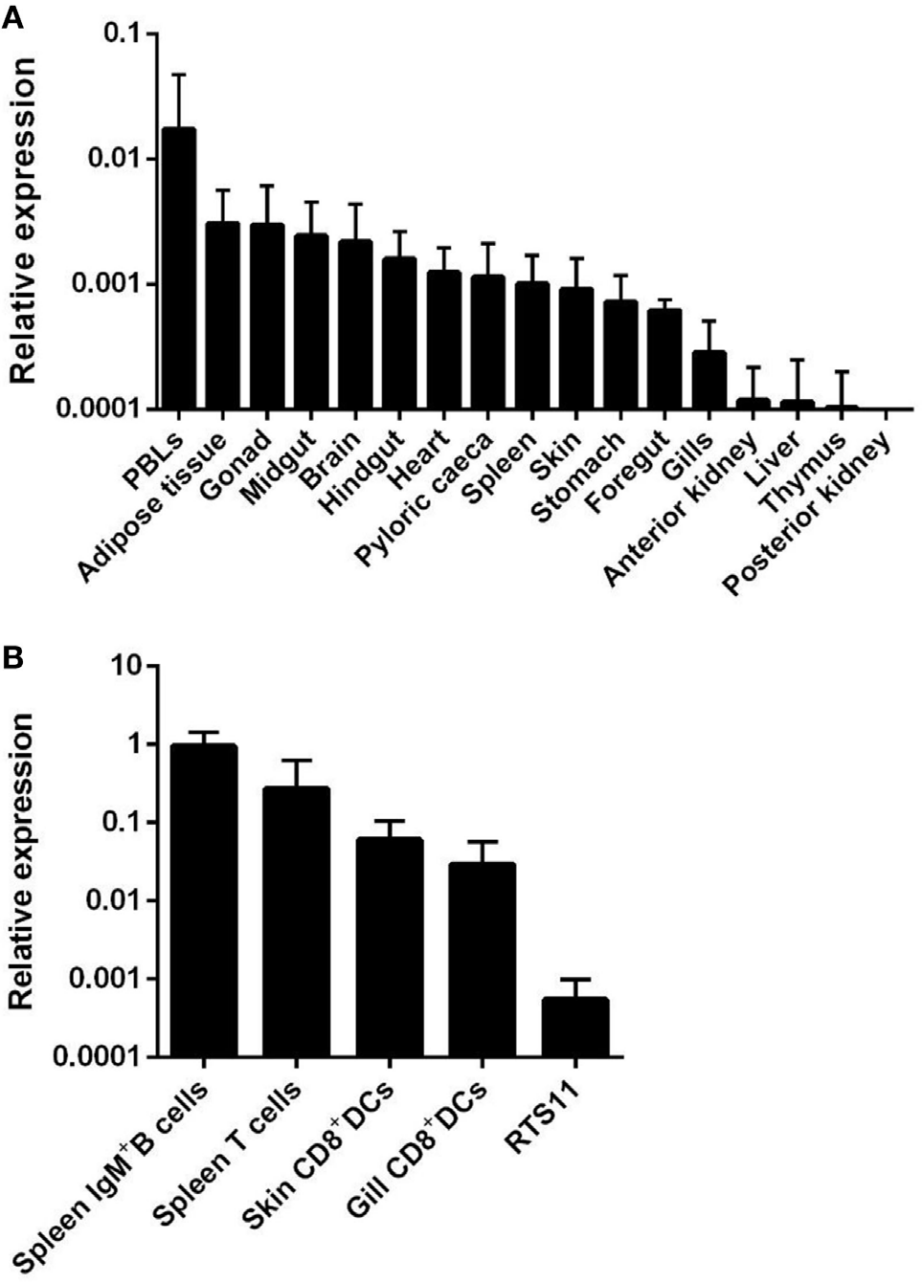
Constitutive transcription levels of APRIL in rainbow trout. **(A)** APRIL mRNA levels in peripheral blood leukocytes and tissues from three naïve perfused fish were estimated by real-time PCR in duplicate samples. **(B)** APRIL transcription levels were also analyzed in FACS isolated leukocyte subsets and compared with those obtained in the RTS11 monocyte–macrophage cell line (*n* = 5). Data are shown as the gene expression relative to the expression of endogenous control EF-1α (mean + SD).

When we analyzed the transcription of APRIL in different tissues obtained from unstimulated perfused rainbow trout, we observed that APRIL mRNA levels were higher in PBLs, followed by adipose tissue, gonad, midgut, brain, hindgut, heart, pyloric caeca, spleen, skin, stomach, foregut, and gills (Figure 1A). On the other hand, constitutive APRIL transcription levels were very low in anterior kidney, liver, thymus, and posterior kidney (Figure 1A).

APRIL transcription levels were also studied in different leukocyte subsets including IgM^+^ B cells and T cells from spleen, skin, and gill CD8^+^ DCs and RTS11 cells. Interestingly, higher APRIL transcription levels were observed in splenic IgM^+^ B cells and T cells obtained from unstimulated fish, while intermediate mRNA levels were observed in both DC subsets (Figure 1B). Lower levels of APRIL transcription were detected in the RTS11 monocyte–macrophage cell line (Figure 1B).

**Figure 2:**
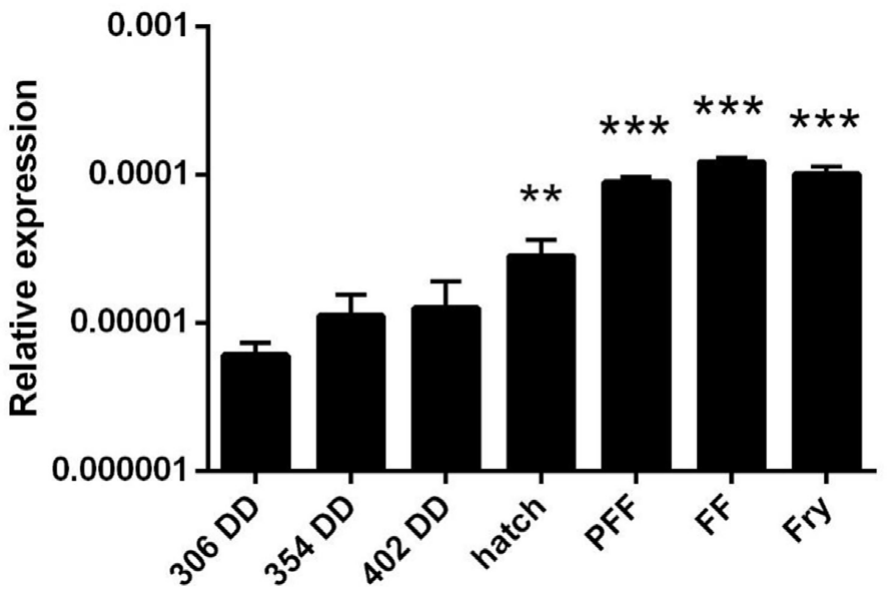
APRIL transcription levels in early rainbow trout developmental stages. Transcriptional levels of APRIL during trout early development at different stages were estimated by real-time PCR. Data are shown as the gene expression relative to the expression of an endogenous control (EF-1α) (mean + SD, *n* = 5). Abbreviations: DD, degree days; HAT, hatching; PFF, 1 week before first feeding; FF, first feeding; Fry, 3 weeks post-first feeding. Asterisks indicate levels of expression significantly different to those observed in samples taken at 306 DD (**p ≤ 0.01 and ***p ≤ 0.005).

Finally, we also analyzed APRIL transcription through the early rainbow trout developmental stages. APRIL transcription was detected in all early developmental stages from 306 DD post-fertilization (Figure 2). Interestingly, a significant increase in APRIL mRNA levels was observed from hatching (HAT) when compared with the APRIL transcription values obtained at 306 DD post-fertilization (Figure 2). This increase was maintained during the posterior stages (Figure 2). These results suggest that APRIL could be playing an important role in the development of the immune system as it has been demonstrated in several teleost species that lymphocytes differentiate within the lymphoid organs around the time of HAT (34).

### APRIL Binds Splenic IgM^+^ B Cells

**Figure 3:**
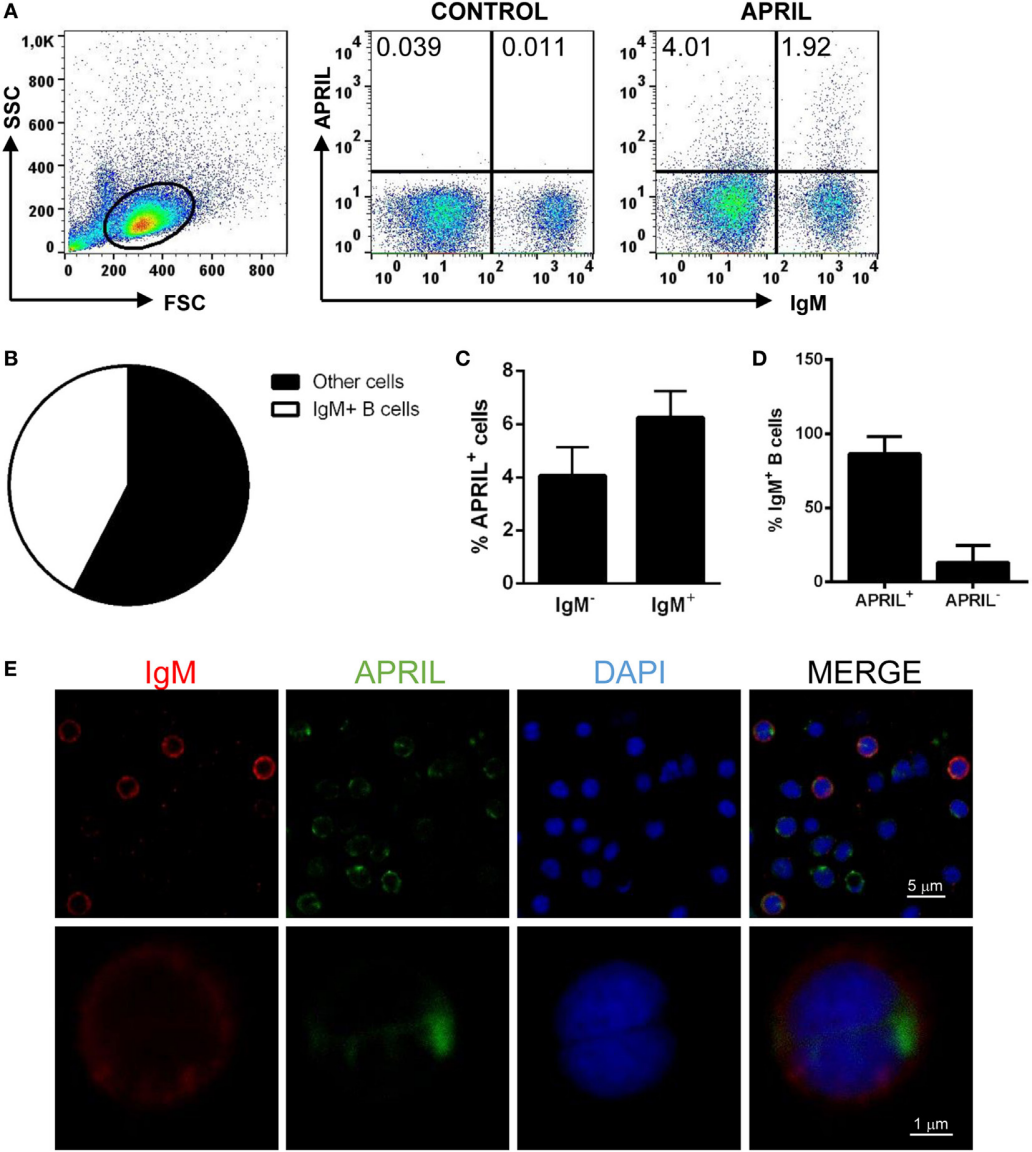
Rainbow trout APRIL binds to IgM^+^ B cells. To analyze the specific binding of APRIL to IgM^+^ B cells, freshly isolated splenic leukocytes were incubated with biotinylated APRIL (1 µg/ml) or with control medium for 15 min. Thereafter, cells were stained with an anti-IgM mAb (1.14 mAb mouse IgG1 coupled to allophycocyanin) together with FITC-streptavidin and analyzed by flow cytometry. Dot plots from one representative experiment are shown **(A)**, along with a pie chart depicting the percentage of IgM^+^ and IgM^−^ cells among APRIL-binding cells **(B)**. The quantification of APRIL-binding cells within the total IgM^−^ and IgM^+^ compartments is also shown **(C)** as mean + SD (*n* = 6). **(D,E)** Total leukocytes from spleen were incubated with recombinant biotinylated APRIL (1 µg/ml) for 15 min, then plated onto poly-l-lysine-coated glass slides, fixed and labeled with anti-IgM (shown as red) and FITC-streptavidin (APRIL, shown as green), then counterstained with DAPI (blue), and analyzed by confocal fluorescence microscopy. Percentages of IgM^+^BAFF^−^ and IgM^+^BAFF^+^ cells on preparations of spleen leukocytes from three fish were quantified and plotted **(D)**, shown as mean + SD (*n* = 500 cells). A representative general overview is shown [**(E)**, upper row] (scale bar, 5 µm) and the amplification detail of a single cell [**(E)**, lower row] (scale bar, 1 µm).

Recombinant APRIL was produced under denaturing conditions, and proper refolding was verified performing PAGE under reducing and non-reducing (native) conditions. The fact that dimers and trimers were visualized when a native electrophoresis was carried out indicated that APRIL was properly folded (Figure S1 in Supplementary Material) (1). Prior to studying the effect of APRIL on rainbow trout IgM^+^ B cells, we studied the capacity of APRIL to bind to rainbow trout IgM^+^ B cells from spleen. In this and all other flow cytometry experiments described throughout the paper, doublets and dead cells were excluded from the analysis following the gating strategy described in Figure S2 in Supplementary Material. An average 5.72 ± 1.14% of live splenocytes bound to recombinant APRIL (Figure 3A), while only an insignificant binding was observed when the C-His protein was used (Figure S3 in Supplementary Material). Surprisingly, most of these APRIL-binding cells (64.7 ± 5.3%) were IgM^−^ cells, while the remaining 35.3 ± 5.3% were IgM^+^ B cells (Figure 3B). Within the IgM^+^ compartment, only an average 6.24 ± 1% of the cells had the capacity to bind APRIL (Figure 3C), thus suggesting that only a minor subset of IgM^+^ B cells in the spleen have the capacity to bind APRIL in physiological conditions. Given that the percentage of B cells that bind APRIL in homeostasis is much higher (35), we decided to examine whether the low percentage of IgM^+^ B cells with attached APRIL on the surface observed though flow cytometry was a result of the rapid internalization of the cytokine inside the cells. For this, we studied the percentage of IgM^+^ B cells that can internalize APRIL by confocal microscopy and in this case we found that an average 86.65 ± 11.5% of the IgM^+^ B cells had internalized APRIL already after 15 min (Figures 3D,E). Thus, most splenic rainbow trout IgM^+^ B cells specifically recognize and internalize APRIL.

### APRIL Increases the Survival of IgM^+^ B Cells and Has Lymphoproliferative Effects

**Figure 4:**
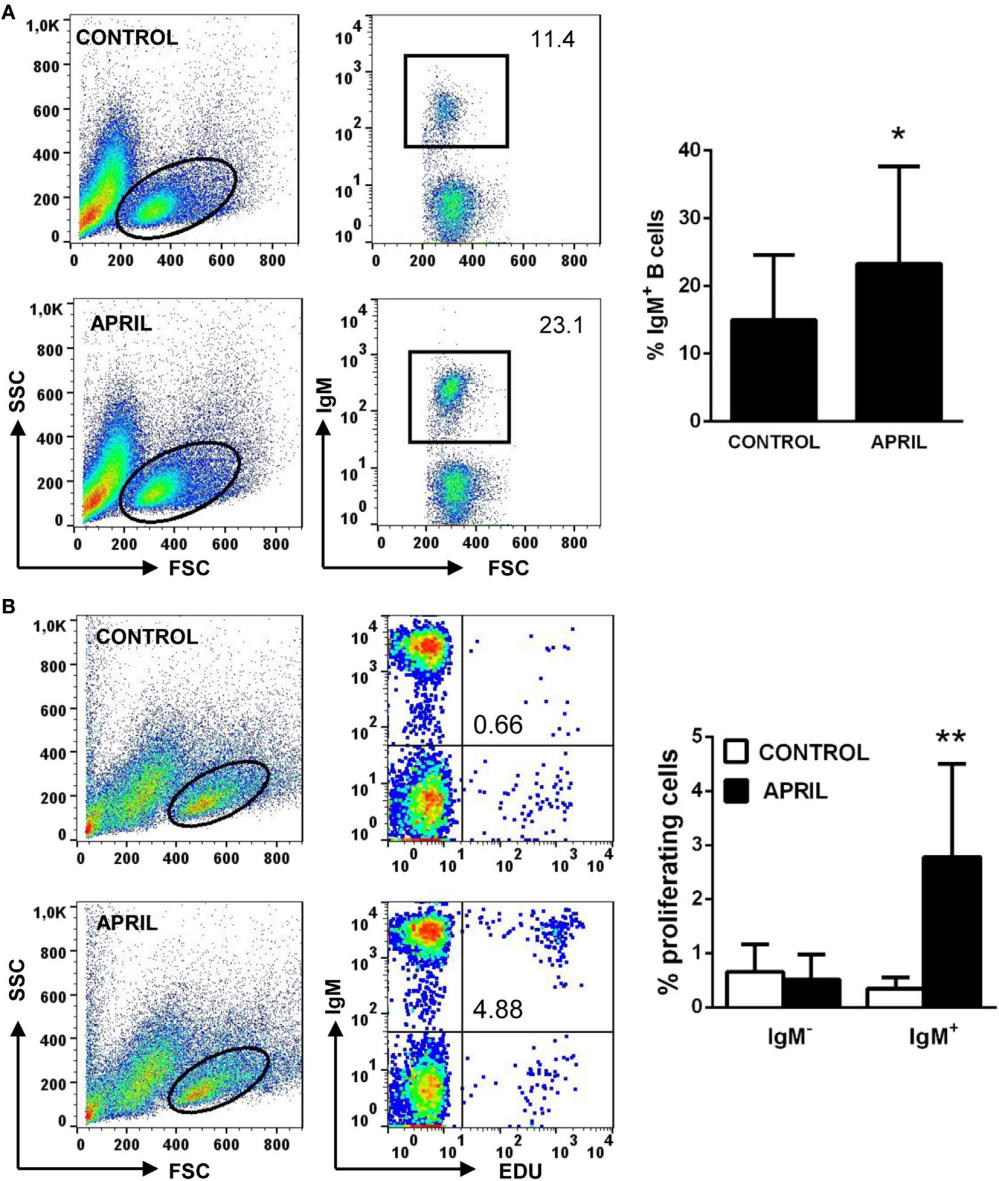
APRIL increases IgM^+^ B cell survival and has lymphoproliferative effects. Spleen leukocytes were incubated with recombinant APRIL (1 μg/ml) or left unstimulated (control). **(A)** After 3 days of incubation at 20°C, cells were labeled with anti-IgM mAb and analyzed by flow cytometry to estimate IgM^+^ B cell survival. Representative plots are shown (left), together with a quantification of average IgM^+^ B cells in cultures (right graph, mean + SD) (*n* = 9). **(B)** In parallel experiments, the lymphoproliferative effects were determined. To carry this out, after 3 days of incubation of splenocytes with APRIL or control media, the cells were labeled with EdU and incubated for a further 24 h. At that point, cells were labeled with anti-IgM mAb and the number of IgM^+^ B cells with incorporated EdU (proliferating cells) determined as described in Section “Materials and Methods.” Representative plots are shown (left), together with a quantification of proliferating IgM^−^ and IgM^+^ cells (right graph, mean + SD) (*n* = 9). Asterisks denote significantly different values in APRIL-treated cultures compared with control cultures (*p ≤ 0.05 and **p ≤ 0.01).

In mammals, APRIL co-stimulates *in vitro* the proliferation of primary B cells (14). Thus, we tested the effect of rainbow trout APRIL on the survival and proliferation of splenic IgM^+^ B cells. We observed that incubation of the splenocytes with APRIL significantly increased the survival of IgM^+^ B cells in these cultures after 3 days (Figure 4A). Thus, our next step was to establish if this increased survival was a consequence of lymphoproliferative effects of APRIL. Our results clearly show that APRIL has a strong capacity to induce the proliferation of splenic IgM^+^ B cells (Figure 4B). These lymphoproliferative effects of APRIL were specific for IgM^+^ B cells, as no proliferation of IgM^−^ B cells was ever observed in these cultures (Figure 4B). No effects of the C-His protein tested in parallel were observed on splenocyte survival or proliferation (data not shown).

### APRIL Increases the Number of Antibody-Secreting Cells

**Figure 5:**
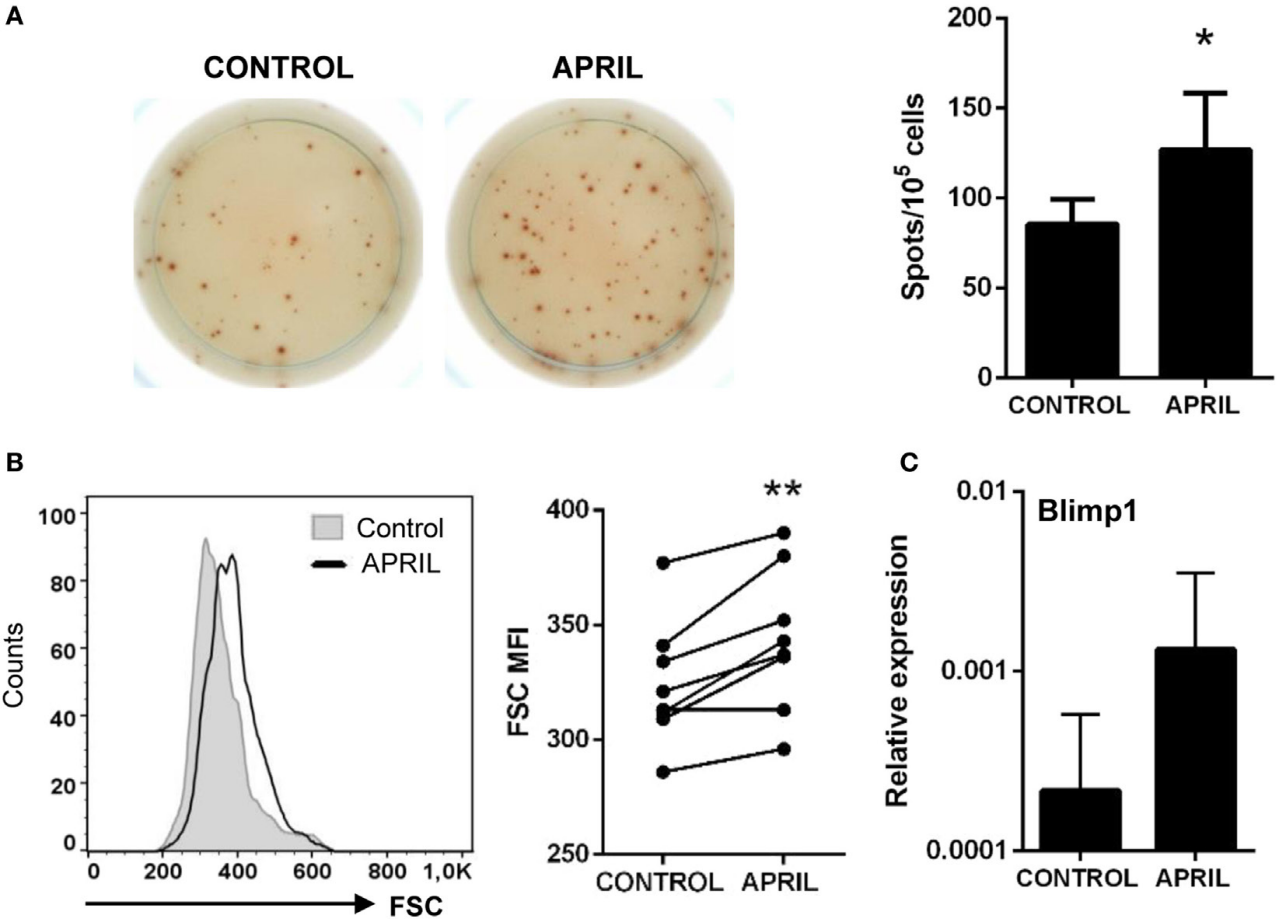
APRIL increases the number of IgM-secreting cells. Splenocytes were incubated with APRIL (1 μg/ml) or media alone for 48 h and then plated in ELISPOT plates previously coated with anti-IgM mAb, for a further 24 h. After incubation, cells were washed away and a biotinylated anti-trout IgM mAb used to detect number of spot forming cells. **(A)** Images from a representative experiment are shown together with a quantification of spot-forming cells, as mean + SD (*n* = 7). **(B)** After 72 h of stimulation with APRIL, splenocytes were labeled with anti-trout IgM mAb and analyzed by flow cytometry. IgM^+^ B cells were gated and the mean fluorescence intensity (MFI) for their forward scatter (FSC) determined. Representative histogram and FSC MFI values for different individual fish under control or APRIL conditions are shown (*n* = 7). **(C)** Splenocyte cultures were treated with APRIL (1 µg/ml) or left unstimulated for 24 h, and then RNA from IgM^+^ FACS isolated B cells was extracted as described in Section “Materials and Methods.” The transcription of Blimp-1 relative to the endogenous control EF-1α was calculated for each sample, and shown as mean + SD. Asterisks denote significantly different values in APRIL-treated cultures compared with control cultures (*p ≤ 0.05 and **p ≤ 0.01).

We performed an ELISPOT to establish whether APRIL could influence IgM secretion in splenocyte cultures. Through this technique, we found that APRIL significantly increased the number of IgM-secreting cells in splenocyte cultures after 3 days (Figure 5A). No effect of the C-His protein on the number of IgM-secreting cells was observed (data not shown). This increase in the number of IgM-secreting cells observed in response to APRIL could be due to an stimulatory effect on spontaneous IgM secretion by non-differentiated B cells, to a differentiation of IgM^+^ B cells to plasmablasts/plasma cells or a could be a consequence of increased survival of pre-existing plasmablasts/plasma cells in the spleen in the presence of APRIL. An activation process of B cells to plasmablasts would imply additional changes in the phenotype of these cells, such as increased size; however, in case, these cells were to differentiate to plasma cells the expression of some transcription factors specific for terminally differentiated plasma cells such as Blimp1 (36) should be increased. In our experiments, we found that IgM^+^ B cells significantly increased their size in response to APRIL (Figure 5B); however, Blimp1 transcription levels were not significantly increased in FACS isolated IgM^+^ B cells from stimulated cultures in comparison to B cells obtained from non-stimulated cultures (Figure 5C). Although an increase in mean Blimp1 transcription seemed apparent, upregulations were not consistently found in all individuals and in these conditions differences were not significant (*p* = 0.251). Taking into account that no increased survival of pre-existing plasmablasts/plasma cells (IgM^+^IgD^−^ B cells) seemed apparent in our cultures (data not shown), our results suggest that APRIL activates IgM secretion in B cells without terminally differentiating them to plasma cells.

### APRIL Increases MHC II Surface Expression in IgM^+^ B Cells

**Figure 6:**
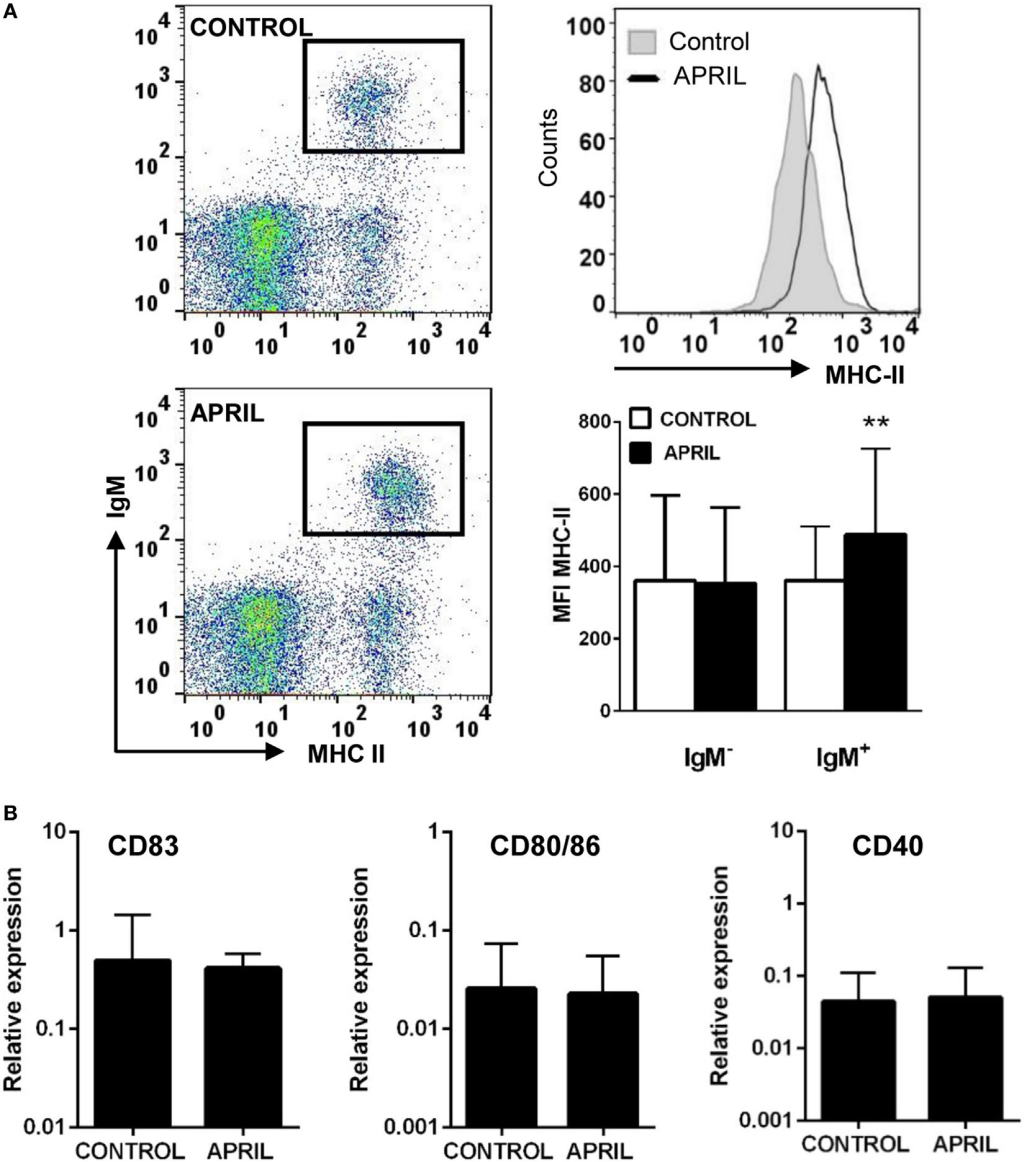
APRIL increases the expression of surface MHC II on IgM^+^ B cells. **(A)** Spleen leukocytes were incubated with APRIL (1 μg/ml) or with control media during 72 h at 20°C. Thereafter, cells were labeled with anti-trout IgM and anti-trout MHC II mAbs and analyzed by flow cytometry (left dot plots). An histogram showing MHC II expression levels in IgM^+^ B cells from one representative fish (upper right) is shown together with a quantification of MHC II mean fluorescence intensity values in IgM^+^ B cells and IgM^−^ cells (lower right), shown as mean + SD (*n* = 9). **(B)** Splenocyte cultures were treated with APRIL (1 µg/ml) or left unstimulated for 24 h, and then RNA from FACS isolated IgM^+^ B cells was extracted as described in Section “Materials and Methods.” The transcription of CD83, CD80/86, and CD40 relative to the endogenous control EF-1α was calculated for each sample, and shown as mean + SD (*n* = 7). Asterisks denote significantly different values in APRIL-treated cultures compared with control cultures (**p ≤ 0.01).

Given their professional antigen-presenting cell nature, B cells constitutively express MHC II on the cell surface (37). Thus, we next studied whether APRIL affected the levels of MHC II surface expression on rainbow trout splenic IgM^+^ B cells. Our results clearly show that APRIL significantly increased the levels of surface MHC II on IgM^+^ B cells (Figure 6A). This effect was not visible when the C-His protein was used (data not shown). However, this increased MHC II expression did not correlate with changes in the transcription of co-stimulatory molecules such as CD40, CD83, or CD80/86 (Figure 6B). Interestingly, this increase in surface MHC II levels was not visible in IgM^−^ cells carrying MHC II on the cell surface (Figure 6A), which would mainly account for the other main subset of B cells found in rainbow trout spleen, IgT^+^ B cells (38).

### APRIL Increases the Capacity of IgM^+^ B Cells to Process Antigens

**Figure 7:**
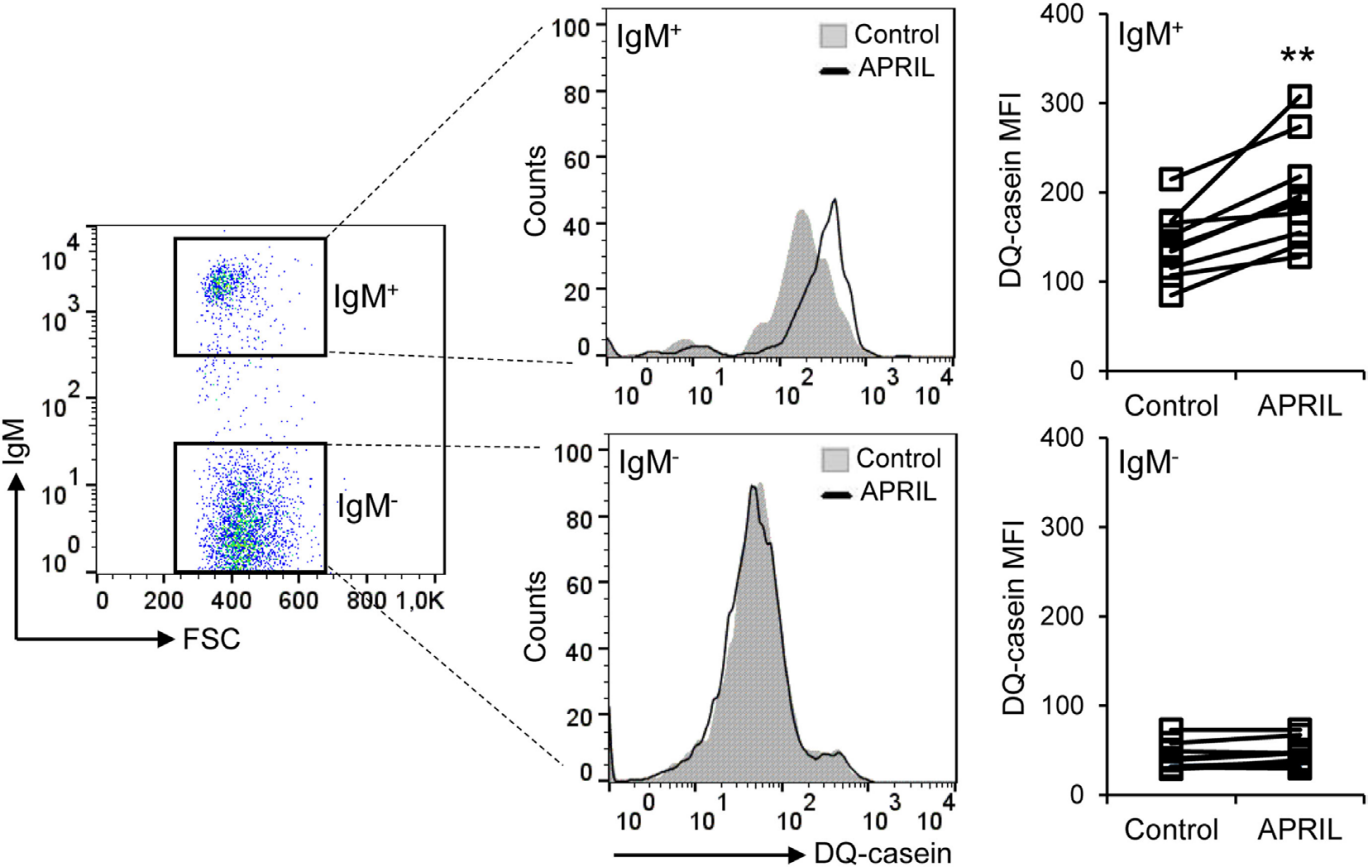
APRIL increases the antigen processing capacity of IgM^+^ B cells. Spleen leukocytes were incubated with APRIL (1 μg/ml) or with control media during 72 h at 20°C. Thereafter, cells were incubated with DQ-CASEIN (5 μg/ml) for 2 h at 20°C. After this incubation, cells were labeled with an anti-trout IgM mAb and analyzed by flow cytometry (left). IgM^+^ (top panels) and IgM^−^ (bottom panels) cells were gated and the signal intensity quantified. Representative histograms are shown, along with DQ-CASEIN mean fluorescence intensity values of IgM^+^ B cells and IgM^−^ cells from different individual fish under control or APRIL conditions (*n* = 10). Asterisks denote significantly different values in APRIL-treated cultures than in control untreated cultures (**p ≤ 0.01).

In both mammals and fish B cells, the antigens acquired through the BCR are processed and their related peptides presented in the context of MHC II on the cell surface (37). Since teleost IgM^+^ B cells are phagocytic, they exert an increased capacity to present not only soluble but also particulate antigens (39). In this context, and given the fact that APRIL seemed to upregulate MHC II surface expression on IgM^+^ B cells, we next examined whether this cytokine was capable of affecting the capacity of B cells to degrade DQ-CASEIN upon endocytosis, a self-quenched form of fluorescently labeled CASEIN, commonly used to study protease-mediated antigen-processing capacities also in fish (40). Due to the positive effects of APRIL on IgM^+^ B cell survival and proliferation, an increased percentage of IgM^+^ B cells processing DQ-CASEIN was observed in splenocyte cultures treated with APRIL in comparison to untreated cultures (Figure 7). However, we also found that the mean fluorescence intensity (MFI) for DQ-CASEIN was higher in IgM^+^ B cells from APRIL-treated cultures than that of the cells in control untreated cultures (Figure 7). This increase was not observed when cultures were treated with the C-His protein instead of APRIL (data not shown). Interestingly, this effect of APRIL on antigen processing was not detected in IgM^−^ cells. Altogether, these results clearly demonstrate that APRIL regulates the antigen processing capacities of IgM^+^ B cells exclusively, having no effects on other B cell populations present in the spleen.

## Discussion

In mammals, it has been hypothesized that the availability of APRIL and/or BAFF at different anatomic sites, coupled with the different signature BAFF/APRIL receptor expression patterns on different B cell subsets found at these sites, strongly conditions how BAFF and APRIL regulate the homeostasis of distinct B cell subsets in an individual (41). In this sense, although teleost fish constitute the first animal group in which all the basic components of adaptive immunity are found, fish B cells share many phenotypical and functional characteristics with mammalian B1 cells, in contrast to mammalian conventional B cells, also referred to as B2 cells. B1 cells, considered elements of the innate immune system, are responsible for the production of natural serum antibodies (42). These antibodies have low affinity and a wide reactivity and are spontaneously produced or are induced in response to TI antigens to interfere with pathogen replication during the initial stages of infection, until a specific B2 TD response is mounted (43). Thus, similar to mammalian B1 cells, fish B cells have been shown to express a wide range of innate receptors that would allow them to immediately sense pathogens (30) and have a strong phagocytic capacity (44, 45). As a consequence, fish B cells are one main cell types implicated in the early response to inflammation (25, 46). Furthermore, fish B cells have a similar surface IgM/IgD ratio, size, and complexity than mammalian B1 cells (47). In addition, unlike mammalian B2 cells and similar to B1 cells, fish IgM^+^ B cells are incapable of proliferating upon BCR cross-linking (47). In this context, it seems interesting to establish how cytokines of the TNF ligand superfamily affect fish B cells, taking into account that B2 and B1 cells seem to respond differently in mammals to these cytokines (35, 41).

Before undertaking functional studies, we analyzed the levels of APRIL mRNA found in homeostasis in different tissues, FACS isolated leukocyte subsets and throughout the early stages of development, as not many APRIL transcriptional studies have been performed to date in teleost fish. In grass carp, the highest APRIL mRNA levels were found in skin, spleen, and anterior kidney (20). However, in rainbow trout, we have seen that anterior kidney APRIL transcription levels are much lower than those observed in other tissues such as adipose tissue, PBLs, spleen, or different segments of the digestive tract. These differences might imply a divergence in the role that APRIL plays in different fish species, which would require the analysis of APRIL functionality in each fish species. Interestingly, BAFF transcription levels are high both in rainbow trout anterior kidney and spleen (24), suggesting that, in this species, BAFF plays a preferential role in B cell development in adult fish taking into account that the anterior kidney is the main hematopoietic site in teleost fish and the place where B cell development takes place (48). Concerning the specific leukocytes subsets that transcribe APRIL and therefore seem to be involved in its production, we have detected APRIL mRNA in skin and gills CD8^+^ DCs, splenic IgM^+^ B cells, and splenic T cells. In mammals, APRIL is known to be produced by different subsets of DCs to regulate B cell responses in the absence of T cell help (49). However, much less is known about which subsets of B cells produce APRIL and the effects that this production has on B cell functionality. In mice, for instance, pro-B cells, pre-B cells, and immature B cells express APRIL mRNA. However, APRIL mRNA is no longer detectable in mature B2 cells, even though mature B1 cells isolated from the peritoneal cavity do express APRIL mRNA at very high levels (9). On the other hand, *in vitro* activation of B2 cells with different stimuli induces the transcription of APRIL (9). Finally, in mammals, APRIL is only transcribed by activated and not by resting Th cells (8). Of course given that our fish come from a fish farm where they might have been exposed to different microbes, we cannot exclude that the B and T cells that are transcribing APRIL in rainbow trout are previously activated cells.

In this study, we have also performed an extensive study to determine the effects of APRIL on splenic B cells for the first time in teleost fish. We have performed this study in splenic B cells because the spleen is the main secondary immune organ in teleosts due to the lack of lymph nodes in this animal group. Our results have shown that APRIL not only increases the survival of trout splenic IgM^+^ B cells, but exerts clear lymphoproliferative effects similar to those observed in response to B cell polyclonal activators such as LPS (50). These results contrast with those obtained with BAFF, as BAFF increases the survival of splenic IgM^+^ B cells in the absence of B cell proliferation (24) and diverge from those obtained in mammalian B cells, where APRIL acts mainly as a co-stimulator of proliferation together with BCR cross-linking (14, 51). Interestingly, experiments performed in mammals with peritoneal B1 cells demonstrated that APRIL, but not BAFF, is the main cytokine responsible for peritoneal cavity B1 cell development or maintenance (35). Given the similarity of rainbow trout IgM^+^ B cells and mammalian B1 cells, APRIL could also be the TNF superfamily cytokine playing a prevailing role in the regulation of the IgM^+^ B cell pool in this species. In addition, it is important to highlight that in our proliferation experiments IgM^+^ B cells constituted the only leukocyte subset within the splenocyte culture that proliferated in response to APRIL. Taking into account that IgT^+^ B cells, a unique B cell subset found in teleost (38, 52), account for up to 11.9% of the leukocytes in the spleen (38), it seems evident to conclude that IgT^+^ B cells do not proliferate in response to APRIL. However, it does seem possible that IgT^+^ B cells could be among the cells that bind APRIL in the spleen as some IgM^−^ cells also had the capacity to bind APRIL. Of course, in mammals, T cells are also regulated by APRIL (8), therefore we cannot exclude that some of these APRIL-binding cells found in the rainbow trout spleen are also T cells.

We have also demonstrated that APRIL increases IgM secretion from splenic B cells, as the number of IgM-secreting cells significantly increased. Similarly, in transgenic mice overexpressing APRIL, serum IgM levels, but not IgG levels, are elevated (8). Likewise, human PBLs treated with recombinant APRIL release IgM to the supernatants (53). However, to the best of our knowledge, the mechanisms through which APRIL increases IgM secretion in mammals have not yet been defined. Our experiments in trout have demonstrated that although there is a significant increase in cell size upon APRIL stimulation, Blimp1 is not significantly upregulated and MHC II surface levels do not decrease, thus suggesting that APRIL is not a differentiation factor for trout B cells into plasma cells but only a stimulating factor for IgM production, similar to LPS (50) or BAFF (24). On the contrary, other cytokines such as interleukin 6 have been shown to induce a terminal differentiation of rainbow trout B cells to plasmablasts/plasma cells that imply Blimp1 upregulation and surface MHC II downregulation (50). Interestingly, when the effects of BAFF on IgM secretion were explored in a previous study, the increased IgM synthesis was postulated as a consequence of an increased survival of pre-existing plasmablasts in the rainbow trout spleen (24), but these effects did not seem as evident in response to APRIL (data not shown).

Finally, we have also investigated the effects of APRIL on the antigen-presenting capacities of rainbow trout IgM^+^ B cells, taking into account that antigen presentation is an important aspect of their immune role in mammals, and especially in fish (39). We have demonstrated that APRIL significantly increases the levels of MHC II on the cell surface of IgM^+^ B cells. Similar effects on MHC II surface expression have also been reported in mice in response to APRIL (15) and in rainbow trout upon BAFF stimulation (24). However, in mice, APRIL also upregulated the expression of co-stimulatory molecules such as CD40, CD80, or CD86 (15), while in rainbow trout, the transcription of CD40, CD80/86, or CD83 was not affected by the incubation with the cytokine. Finally, we also studied the effect of APRIL on the capacity to process antigens, as this would also have an effect in its antigen-presenting abilities. Our results clearly show that IgM^+^ B cells have significantly higher antigen-processing capacities when stimulated with APRIL. Of course whether the increased IgM secretion and antigen-presenting capacities are being exerted on the same B cell or in different B cell subsets is something that should be explored in the future as more markers for specific B cell subsets become available. Interestingly, once again, the effects that APRIL exerts on the antigen-presenting capacities (MHC II surface expression and antigen processing) seemed exclusive of IgM^+^ B cells, as they were not regulated by APRIL in MHC II^+^IgM^−^ cells, which would mainly account for IgT^+^ B cells. Hence, it seems evident that APRIL regulates IgT^+^ B cells differently than IgM^+^ B cells and this is something that should be further explored in the future.

In conclusion, our results demonstrate that a large percentage of rainbow trout IgM^+^ B cells in homeostasis have the capacity to bind and rapidly internalize APRIL. This cytokine seems as a key regulator of IgM^+^ B cell functionality in this species. The effects of APRIL on rainbow trout IgM^+^ B cells include induction of proliferation, increase in IgM secretion, and upregulation of the antigen-presenting capacities of splenic IgM^+^ B cells. Interestingly, these effects appeared exclusively on IgM^+^ B cells, suggesting that IgT^+^ B cells are not regulated by APRIL or are regulated in a different way. Taking into account that teleost IgM^+^ B cells share many functional and phenotypical characteristics of mammalian B1 cells which mediate TI responses in mammals (47); and knowing that teleost fish do not produce GCs and therefore respond to antigens extrafollicularly, without having a specific site in which they can interact with T cells (54); it seems plausible to hypothesize that APRIL preferentially regulates TI processes both in mammals and fish through the regulation of early IgM responses.

## Ethics Statement

The experiments described comply with the Guidelines of the European Union Council (2010/63/EU) for the use of laboratory animals and were previously approved by the Ethics committee from the Instituto Nacional de Investigación y Tecnología Agraria y Alimentaria (INIA; Code CEEA PROEX002/17).

## Author Contributions

IS performed most of the experimental work, with help from EM. DM performed the ELISPOT analyses. CT and AG designed the experiments and wrote the main body of the paper, with contributions from IS.

## Conflict of Interest Statement

The authors declare that the research was conducted in the absence of any commercial or financial relationships that could be construed as a potential conflict of interest.
