# Supplementary material for: Regulation of IgM+ B Cell Activities by Rainbow Trout APRIL Reveals Specific Effects of This Cytokine in Lower Vertebrates
Source: Front Immunol. 2018 Aug 13;9:1880. doi: 10.3389/fimmu.2018.01880 (PMC6099200; doi:10.3389/fimmu.2018.01880)
Supplement: Supplementary file 1 [file Presentation_1.pptx]

## Slide 1
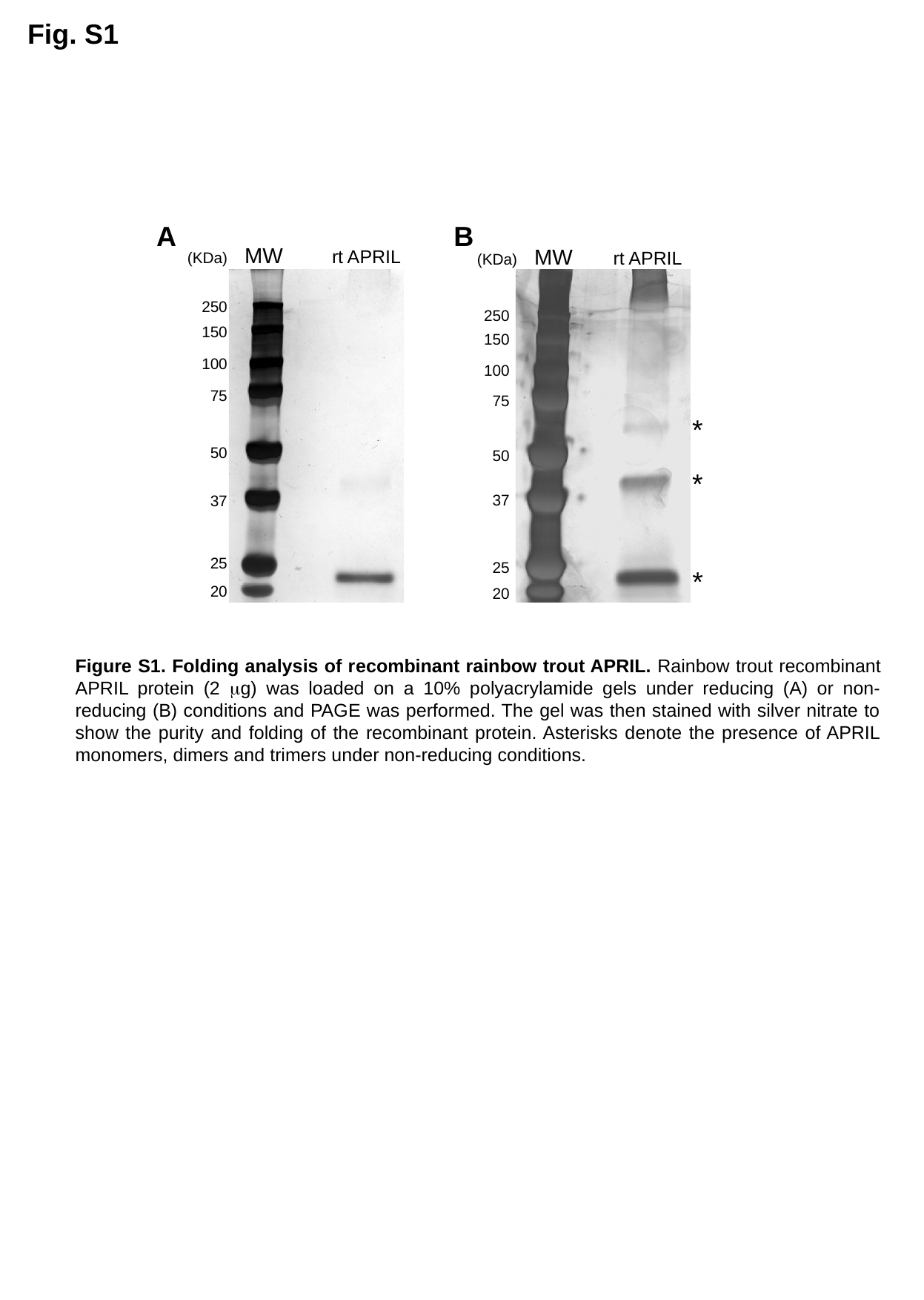

Fig. S1
B
A
(KDa) MW
(KDa) MW
rt APRIL
rt APRIL
250
250
150
150
100
100
75
75
*
50
50
*
37
37
25
25
*
20
20
Figure S1. Folding analysis of recombinant rainbow trout APRIL. Rainbow trout recombinant APRIL protein (2 mg) was loaded on a 10% polyacrylamide gels under reducing (A) or non-reducing (B) conditions and PAGE was performed. The gel was then stained with silver nitrate to show the purity and folding of the recombinant protein. Asterisks denote the presence of APRIL monomers, dimers and trimers under non-reducing conditions.

## Slide 2
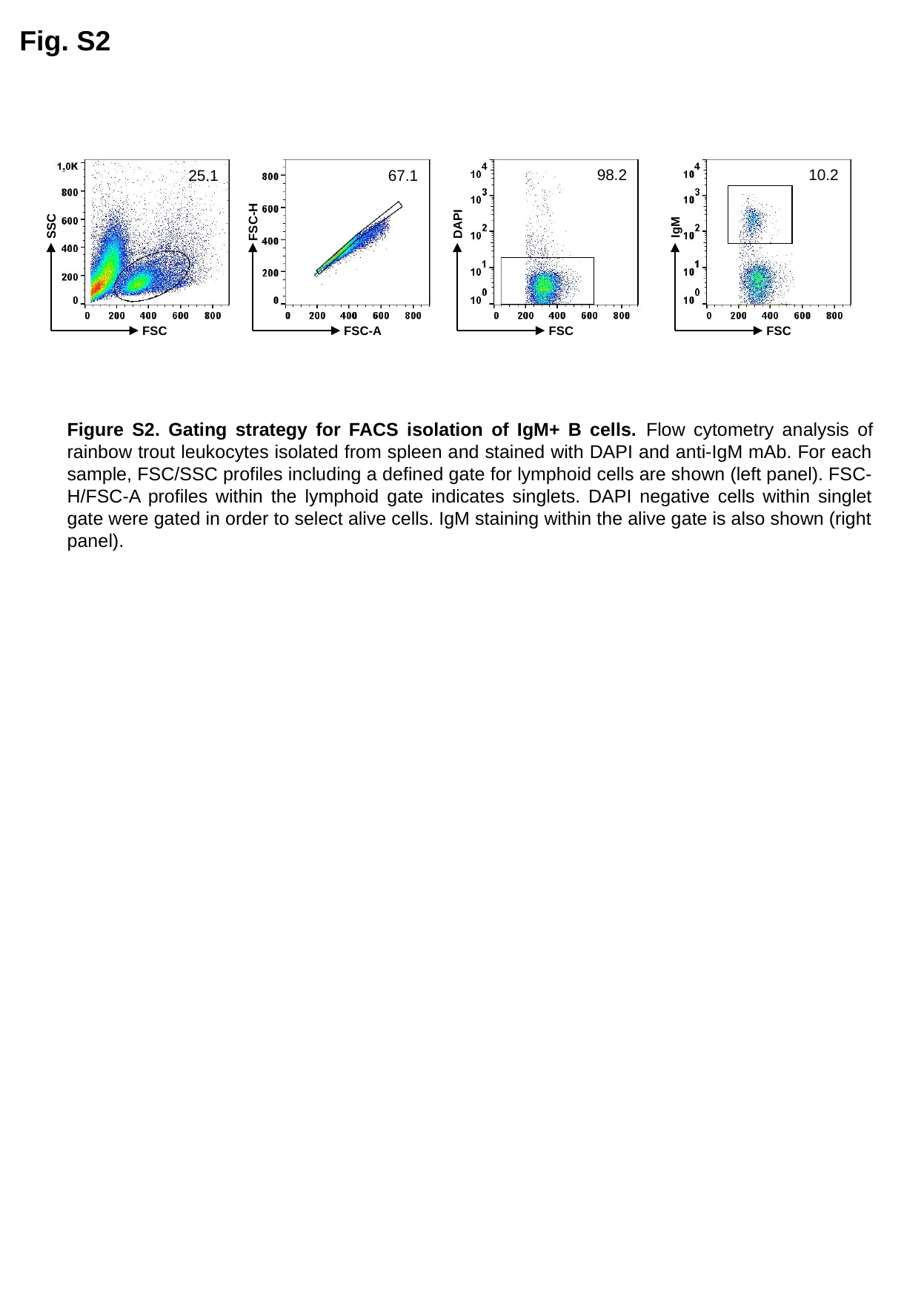

Fig. S2
10.2
98.2
25.1
67.1
FSC-H
DAPI
SSC
IgM
FSC
FSC-A
FSC
FSC
Figure S2. Gating strategy for FACS isolation of IgM+ B cells. Flow cytometry analysis of rainbow trout leukocytes isolated from spleen and stained with DAPI and anti-IgM mAb. For each sample, FSC/SSC profiles including a defined gate for lymphoid cells are shown (left panel). FSC-H/FSC-A profiles within the lymphoid gate indicates singlets. DAPI negative cells within singlet gate were gated in order to select alive cells. IgM staining within the alive gate is also shown (right panel).

## Slide 3
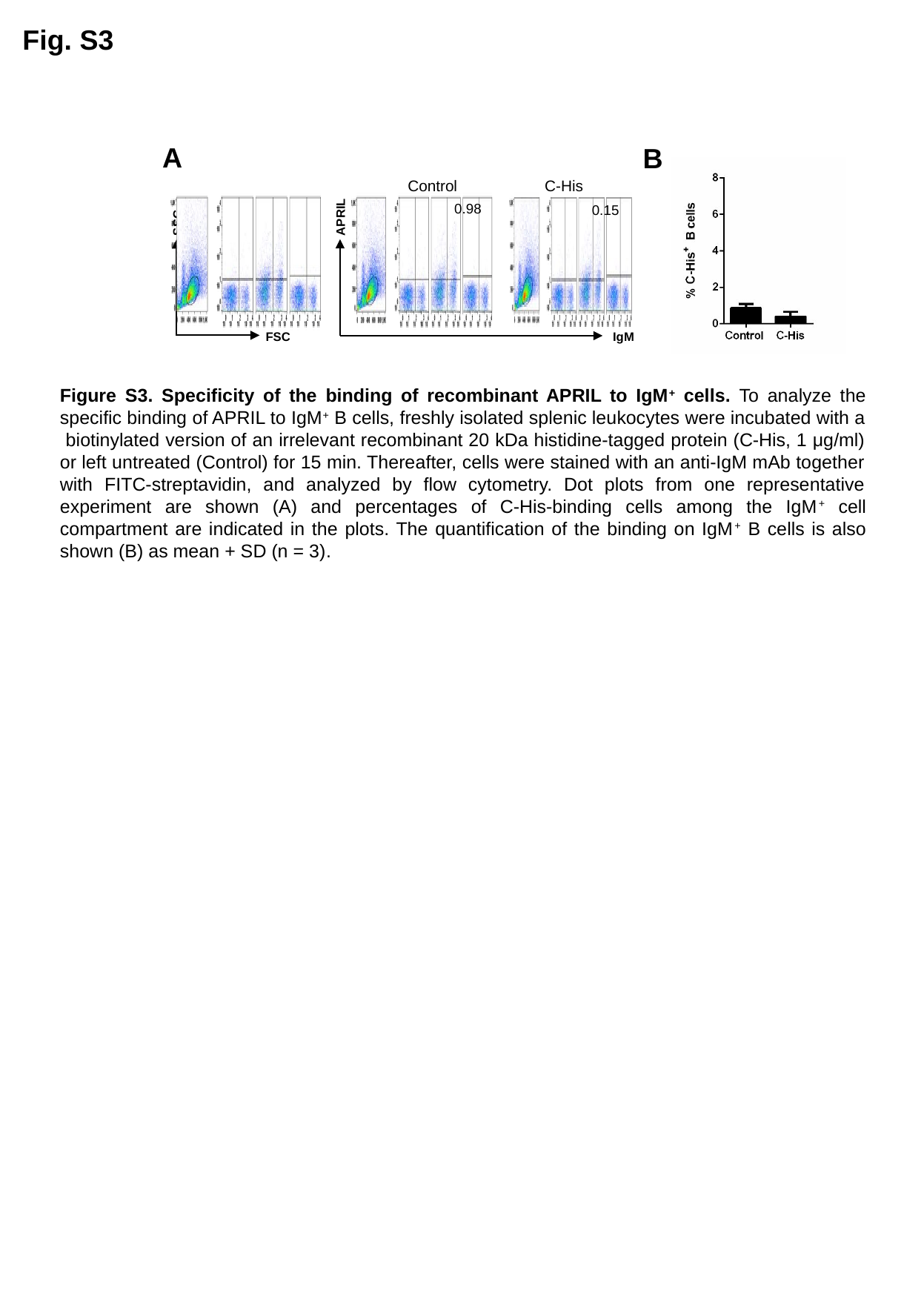

Fig. S3
A
B
Control
C-His
APRIL
IgM
0.98
0.15
SSC
FSC
Figure S3. Specificity of the binding of recombinant APRIL to IgM+ cells. To analyze the specific binding of APRIL to IgM+ B cells, freshly isolated splenic leukocytes were incubated with a biotinylated version of an irrelevant recombinant 20 kDa histidine-tagged protein (C-His, 1 μg/ml) or left untreated (Control) for 15 min. Thereafter, cells were stained with an anti-IgM mAb together with FITC-streptavidin, and analyzed by flow cytometry. Dot plots from one representative experiment are shown (A) and percentages of C-His-binding cells among the IgM+ cell compartment are indicated in the plots. The quantification of the binding on IgM+ B cells is also shown (B) as mean + SD (n = 3).

## Slide 4
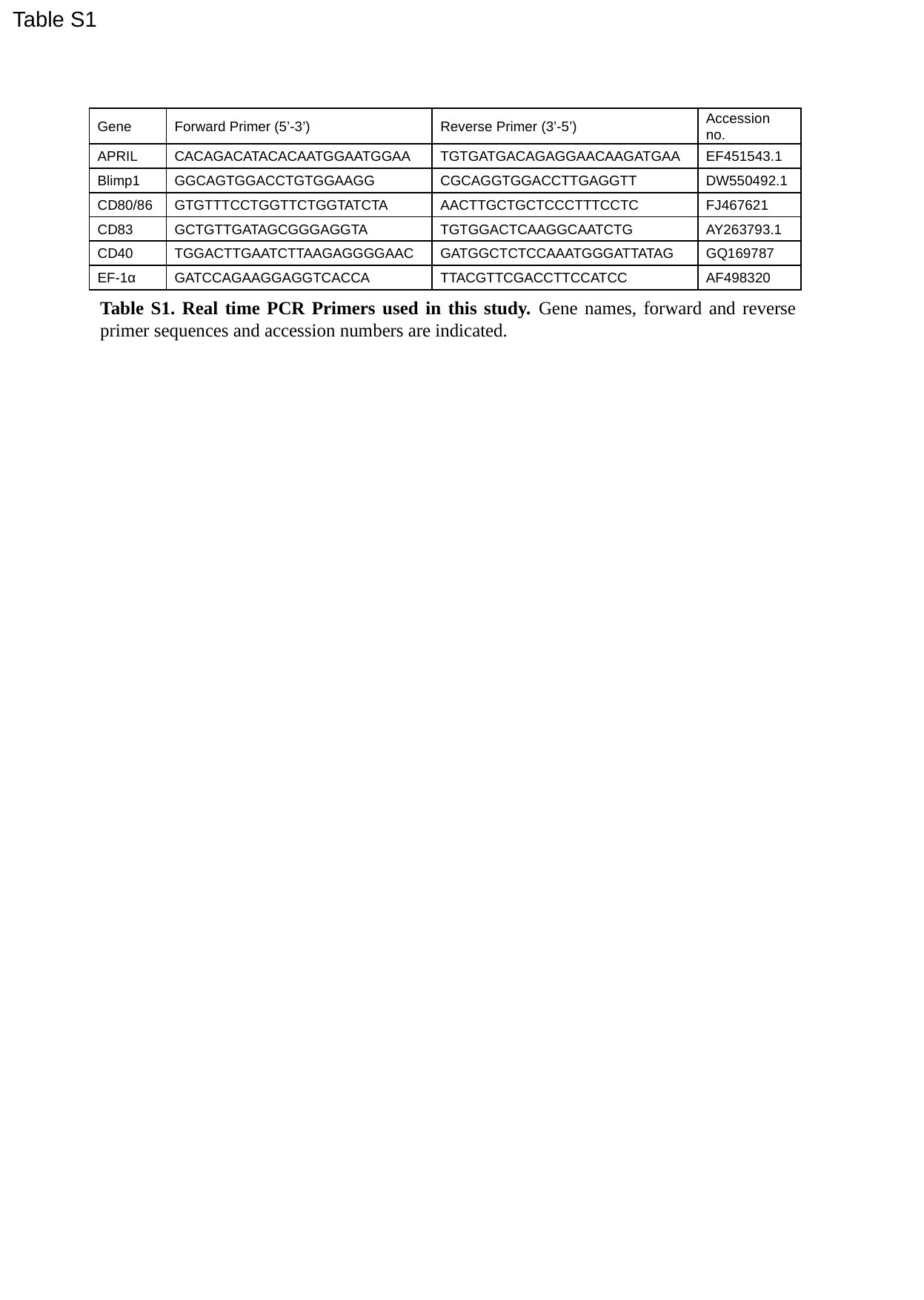

Table S1
| Gene | Forward Primer (5’-3’) | Reverse Primer (3’-5’) | Accession no. |
| --- | --- | --- | --- |
| APRIL | CACAGACATACACAATGGAATGGAA | TGTGATGACAGAGGAACAAGATGAA | EF451543.1 |
| Blimp1 | GGCAGTGGACCTGTGGAAGG | CGCAGGTGGACCTTGAGGTT | DW550492.1 |
| CD80/86 | GTGTTTCCTGGTTCTGGTATCTA | AACTTGCTGCTCCCTTTCCTC | FJ467621 |
| CD83 | GCTGTTGATAGCGGGAGGTA | TGTGGACTCAAGGCAATCTG | AY263793.1 |
| CD40 | TGGACTTGAATCTTAAGAGGGGAAC | GATGGCTCTCCAAATGGGATTATAG | GQ169787 |
| EF-1α | GATCCAGAAGGAGGTCACCA | TTACGTTCGACCTTCCATCC | AF498320 |
Table S1. Real time PCR Primers used in this study. Gene names, forward and reverse primer sequences and accession numbers are indicated.
